# Gluten-free diet adherence patterns and health outcomes in celiac disease: a retrospective observational study

**DOI:** 10.1186/s12876-025-04193-3

**Published:** 2025-08-18

**Authors:** Saba Rahimi, Mohadeseh Mahmoudi Ghehsareh, Nastaran Asri, Mehdi Azizmohammad Looha, Somayeh Jahani-Sherafat, Carolina Ciacci, Mohammad Rostami-Nejad

**Affiliations:** 1 Celiac Disease and Gluten Related Disorders Research Center, Research Institute for Gastroenterology and Liver Diseases, Shahid Beheshti University of Medical Sciences, Tehran, Iran; 2 Gastroenterology and Liver Diseases Research Center, Research Institute for Gastroenterology and Liver Diseases, Shahid Beheshti University of Medical Sciences, Tehran, Iran; 3 Basic and Molecular Epidemiology of Gastrointestinal Disorders Research Center, Research Institute for Gastroenterology and Liver Diseases, Shahid Beheshti University of Medical Sciences, Tehran, Iran; 4 Laser Application in Medical Sciences Research Center, Shahid Beheshti University of Medical Sciences, Tehran, Iran; 5 Department of Medicine, Surgery and Dentistry, Scuola Medica Salernitana, University of Salerno, Salerno, Italy

**Keywords:** Celiac disease, Follow-up, Gluten-free diet, Gastrointestinal symptoms, Non-gastrointestinal symptoms

## Abstract

**Background:**

Celiac disease (CeD) is an autoimmune disorder, causing significant gastrointestinal (GI) and non-GI symptoms. The only effective treatment is a gluten-free diet (GFD), but adherence can be challenging. This study investigates GFD adherence among CeD patients and the change in their clinical and pathological conditions over time.

**Methods:**

A telephone-based retrospective observational analysis conducted between November 2022 and June 2023 enrolled 300 participants with confirmed CeD, selected via systematic sampling from an initial cohort of 1,268 individuals. A comprehensive questionnaire assessed demographics, clinical symptoms, and GFD adherence. Participants were categorized into short/meidum-term (≤ 6 months), long-term (6–24 months), and very long-term (≥ 24 months) GFD adherence groups. All statistical analyses were conducted using R (version 4.3.1), employing a variety of packages tailored for descriptive and inferential statistics.

**Results:**

Participants’ mean age was 41.58 (± 12.37) years, with females constituting 73.67% (*n* = 221/300). GFD adherence was assessed, revealing that 55.33% (*n* = 166/300) of patients adhered strictly to the diet, while 28.33% (*n* = 85/300) experienced worsening adherence over time (*p* = 0.001). Significant improvements were observed in GI and non-GI symptoms across disease duration, with abdominal pain absence increasing from 71.2 to 84.9% (*p* < 0.01), and depression absence improving from 63.6 to 89.6% (*p* < 0.001). Additionally, laboratory values such as VitD and HCT showed improvement (*p* < 0.05), while the comorbidity burden decreased significantly (*p* = 0.001).

**Conclusions:**

Enhanced patient support, including dietary and mental health resources, is vital for improving outcomes in CeD patients. Further research should explore barriers to adherence and the psychosocial effects of living with CeD.

**Supplementary Information:**

The online version contains supplementary material available at 10.1186/s12876-025-04193-3.

## Background

Celiac disease (CeD) is an autoimmune disorder triggered by the ingestion of gluten, a protein found in wheat, barley, and rye, that causes damage to the structure and function of the small intestine. CeD is considered a significant worldwide public health problem due to its global distribution with a prevalence of about 1% [1, 2]. CeD is commonly accompanied by a range of gastrointestinal (GI) symptoms, including diarrhea, nausea, bloating, constipation, and weight loss, as well as non-GI symptoms like headaches, skin disorders, neurological disorders, and fatigue [3–5]. To date, following a strict lifelong gluten-free diet (GFD) is the only effective treatment for CeD. While most patients respond well, those with refractory disease, poor dietary adherence, or delayed diagnosis remain at higher risk for complications (e.g., malnutrition, osteoporosis, or lymphoma) that impact their overall well-being [6].

GFD adherence can be accompanied by some obstacles including the lack of gluten-free products, their high cost, misleading food labeling, lack of information etc [7–10]. Adolescents with CeD face additional challenges related to the GFD. Participating in events like parties, sleepovers, and dining outside can be tricky for them [10–12].

GFD has numerous benefits such as relieving clinical symptoms, addressing nutritional deficiencies, protecting against malignancy, and decreasing the likelihood of developing other autoimmune diseases associated with CeD [13].

Patients following a GFD, especially those in the early stages of diagnosis, need personalized care and support to help them make informed dietary choices and effectively cope with the challenges of living with CeD. This includes regular follow-up appointments with a gastroenterologist, consultations with general practitioners and physicians, and guidance from a registered celiac dietitian for a supervised GFD plan [14–17].

Regular follow-up is crucial to evaluate the progress of the disease, ensure adherence to the strict GFD, and monitor the quality of life of CeD patients, and their ability to manage the condition [18–20]. By utilizing questionnaires tailored to specific communities, healthcare professionals gain valuable insights into patients’ symptoms and overall well-being [21]. This deeper understanding empowers them to provide appropriate treatment and support for patients [22].

This study employed a structured assessment combining the validated Biagi adherence tool with supplemental questions to evaluate GFD adherence and health outcomes.

## Methods

This study is a telephone-based retrospective observational analysis, relying on data collected at a single time point via patient recall and medical records. Actually, the study collected data at one time point via telephone survey, combining patient recall of current status with medical record abstraction for baseline clinical metrics. This study was conducted from November 2022 to June 2023 and a pre-designed questionnaire was completed for each individual. The questionnaire used in the study has been provided as a supplementary file.

**Fig. 1:**
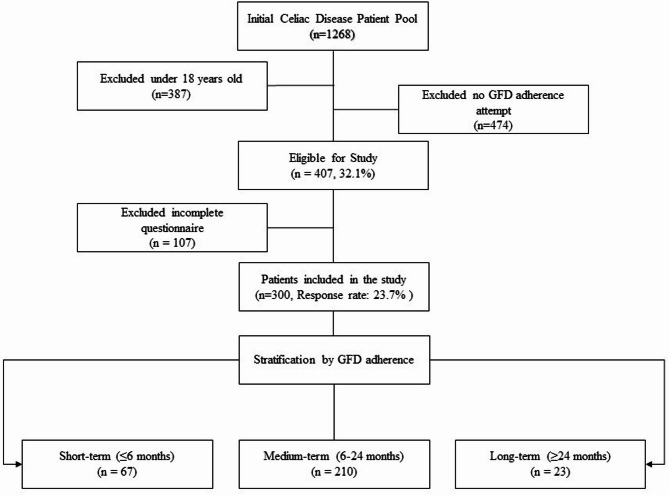
Flow chart showing the basis for selecting the participants

From an initial cohort of 1,268 diagnosed patients, 300 were recruited for this study (23.7% response rate). The inclusion criteria for participants in the current study consisted of being over 18 years old and confirmed diagnosis of CeD according to the American Gastroenterological Association (AGA) recommendations. All cases verified by positive serology (positive anti-tTG IgA and/or EMA IgA) and duodenal biopsy demonstrating Marsh 2–3 histopathology [23]. Patients who did not adhere to the GFD, who were under 18 years old, or who did not complete the questionnaire were excluded from the study (Fig. 1). The patients who did not adhere to the GFD were excluded because they lacked any adherence baseline for comparison, and our study focus was evaluating outcomes among patients making some effort to follow medical advice. Importantly, we retained all patients with any history of attempted adherence, including those who later discontinued. This approach allowed us to study the spectrum from poor to excellent adherence.

The average age of participants was 41.58 years (± 12.37). The majority were female (73.67%) and married (72.67%).

### Data Collection Instruments

Data were collected via a structured telephone survey incorporating the validated 4-item Biagi adherence tool, supplemented with additional questions adapted from clinical follow-up protocols. These supplemental items addressed demographics, symptoms (e.g., GI/non-GI manifestations), and dietary challenges. The questionnaire was reviewed by gastroenterologists and dietitians for clinical relevance and piloted with 20 CeD patients to optimize clarity. Trained interviewers administered the survey using a standardized script to ensure consistency.

The questionnaire assessed GI symptoms (like abdominal pain, diarrhea, bloating), Non-GI symptoms (like depression, anemia), and lab values (like VitD, HCT). Baseline clinical and laboratory data were extracted by the study team from medical records at the time of each patient’s original diagnosis. Current status was assessed via structured phone survey. This dual-source approach minimized recall bias for objective measures. Laboratory values were obtained retrospectively from routine clinical follow-up records at diagnosis and most recent visits; no additional blood tests were conducted specifically for this study.

### GFD adherence evaluation

Patients completed the Biagi adherence questionnaire. This validated tool measures GFD compliance in adults with CeD. The survey comprises four questions, and the questionnaire generates a numerical score, enabling the monitoring of GFD strictness over time [24]. The questionnaire provides a final score categorized into five levels (0–IV), which can be clinically grouped into three levels. Patients scoring 0-I on the Biagi questionnaire represent those with inconsistent or unintentional gluten exposure, whereas excluded non-GFD patients were intentional treatment-refusers with no dietary modification attempts. Patients with scores of II adhere to a GFD, but with significant errors that require correction. On the other hand, patients with scores of III and IV demonstrate strict adherence to a GFD [24]. In addition to assessing GFD adherence through the Biagi questionnaire, we conducted a subjective evaluation of the patients’ opinions regarding their adherence to the diet. Subjective assessments included patient self-evaluation of GFD adherence (good/weak/none), open-ended questions on barriers to adherence, and perceived changes in symptoms since diagnosis.

Patients were stratified by GFD adherence duration into three groups: short/medium-term: ≤6 months, long-term: 6–24 months, and very long-term: ≥24 months.

### Statistical analysis

All analyses were performed using R (v4.3.1). Continuous variables were expressed as mean ± standard deviation (normally distributed) or median with interquartile range (non-normally distributed), while categorical variables were reported as frequencies and percentages. Key outcomes included symptom absence (defined as patient-reported absence of GI and non-GI symptoms), CeD duration groups (categorized as short/medium-term [≤ 6 months], long-term [6–24 months], and very long-term [≥ 24 months]), and comorbidity prevalence (assessed as the count of coexisting conditions from medical records). Group comparisons were performed using ANOVA or Kruskal-Wallis tests for continuous variables and Fisher’s exact tests for categorical variables. Trends over time were evaluated with Wilcoxon signed-rank tests. A two-sided p-value < 0.05 was considered statistically significant.

## Results

### Demographic and lifestyle characteristics of studied CeD patients

This study examined demographic and lifestyle factors impacting disease management. As shown in Table 1, the mean participant age was 41.58 (± 12.37) years old. The majority were female (73.67%), and married (72.67%). Physicians were the primary source of dietary information (27.33%), followed by social media along with physicians (14.67%).

**Table 1:** Demographic and lifestyle factors of studied participants

Variables	Levels	Mean ± SD/Frequency (Percentage)
Age	----	41.58 ± 12.37
Body mass index (BMI) at the time of diagnosis	----	22.88 ± 4.65
Sex	Female	221 (73.67)
	Male	79 (26.33)
Marriage	Single	80 (26.67)
	Married	218 (72.67)
	Widowed	1 (0.33)
	Divorced	1 (0.33)
Diet information source	Social media	15 (5.00)
	Physicians	82 (27.33)
	Books	5 (1.67)
	Question	2 (0.67)
	Social media + Physicians	44 (14.67)
	Social media + Books	6 (2.00)
	Social media + Question	3 (1.00)
	Physicians + Books	43 (14.33)
	Physicians + Question	5 (1.67)
	Social media + Physicians + Books	41 (13.67)
	Social media + Physicians + Question	12 (4.00)
	Social media + Books + Question	1 (0.33)
	Physicians + Books + Question	2 (0.67)
	Social media + Physicians + Books + Question	38 (12.67)

Normally distributed continuous variables were presented as mean ± standard deviation (SD), while non-normal continuous variables were presented as median (interquartile range [IQR]). Categorical variables were described by frequency and percentage

### GFD adherence patterns and behavioral trends in CeD patients

Table 2 presented GFD adherence patterns in the study cohort. Patients were stratified by duration of active GFD adherence: 22.3% (*n* = 67) were short/medium-term adherents, 70% (*n* = 210) long-term, and 1.33% (*n* = 4) very long-term adherents. The median time since initial GFD adoption across all participants was 9 [6, 11] months (range: 1–30 months).

**Table 2:** GFD adherence patterns in CeD patients

Variables	Levels	Median (IQR)/Frequency (Percentage)
GFD duration	-------	9 (6, 11)
	~ 6 months (Short/Medium-Term)	67 (22.33)
	6–24 months (Long-Term)	210 (70.00)
	>=24 months (Very Long-Term)	23 (1.33)
Biagi questionnaire Score	Do not follow (0)	65 (21.66)
	Rarely follows GFD diet (I)	55 (18.33)
	Important Errors (II)	14 (4.66)
	Follow a GFD well (III)	3 (1.00)
	Follow a strict GFD (IV)	163 (54.33)
subjective evaluation of GFD adherence	No adherence	19 (6.33)
	Weak adherence	77 (25.67)
	Good adherence	204 (68.00)
Change in GFD adherence	Becomes poor	85 (28.33)
	Becomes better	87 (29.00)
	Not change	126 (42.00)

Normally distributed continuous variables were presented as mean ± standard deviation (SD), while non-normal continuous variables were presented as median (interquartile range [IQR]). Categorical variables were described by frequency and percentage

By implementing Biagi questionnaire, we aimed to assess GFD adherence among CeD patients. According to the results, out of the 300 patients, 166 (55.33%) scored III or IV, while 14 (4.66%) patients scored II, and 120 (40%) scored I or 0 in terms of GFD adherence.

Upon analyzing the patients’ subjective evaluation of their GFD adherence, we found that 68% demonstrated good adherence, while 25.67% exhibited weak adherence, and 6.33% did not adhere at all (after an initial period of adherence). Moreover, 13% of those who claimed strictly adhere to the GFD actually received a score of II on Biagi questionnaire, which is considered unsatisfactory and has significant errors. Changes in adherence over time indicated that 28.33% became worse, 29.00% improved, and 42.00% remained unchanged.

When we inquired about the underlying causes of the lack of good adherence to the GFD, most participants cited dietary difficulty as the main barrier.

### Improvements in GI and non-GI symptom across CeD duration

**Fig. 2:**
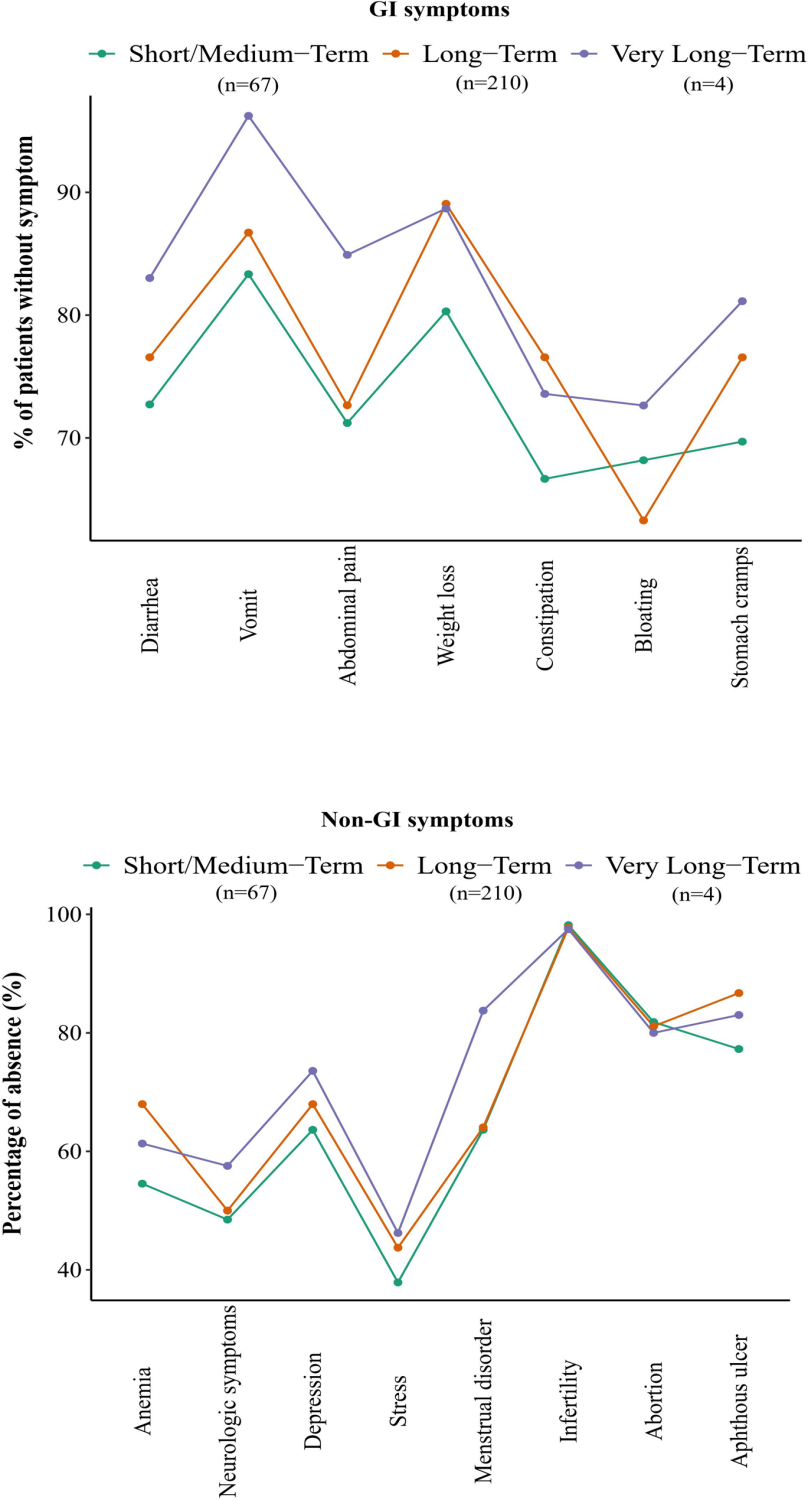
Overall Comparison of GI and Non-GI Symptom Absence by GFD Adherence Duration in CeD Patients

Figure 2 illustrates the relationship between the duration of CeD and the percentage of absence of GI and non-GI symptoms. As the duration of CeD increases from short/medium-term to very long-term, the percentage of absence for most symptoms generally improves. Among GI symptoms, vomiting showed the highest absence percentage in the very long-term group, reaching 96.2%, compared to 83.3% in the short/medium-term group and 86.7% in the long-term group. Similarly, abdominal pain and diarrhea absence also improved with duration, with abdominal pain absence increasing from 71.2% in the short/medium-term group to 84.9% in the very long-term group. Diarrhea absence showed a smaller but noticeable improvement, increasing from 72.7% in the short/medium-term group to 83.0% in the very long-term group. These trends suggest better symptom management with prolonged disease duration. However, some symptoms exhibited less improvement or even slight declines. For example, constipation absence, which was highest in the long-term group (76.6%), decreased slightly in the very long-term group to 73.6%. Similarly, bloating absence remained relatively low across all groups, showing only minor improvement from 68.2% in the short/medium-term group to 72.6% in the very long-term group.

In the context of non-GI symptoms, depression showed the most significant improvement, with absence percentages increasing from 63.6% in the short/medium-term group to 89.6% in the very long-term group. Anemia and neurologic symptoms also demonstrated improvement, with absence rates of 54.5% and 48.5% in the short/medium-term group, rising to 61.3% and 71.7%, respectively, in the very long-term group.

### Changes in Laboratory Values, Comorbidities, and Disorder Prevalence Over Time in CeD Patients

**Fig. 3:**
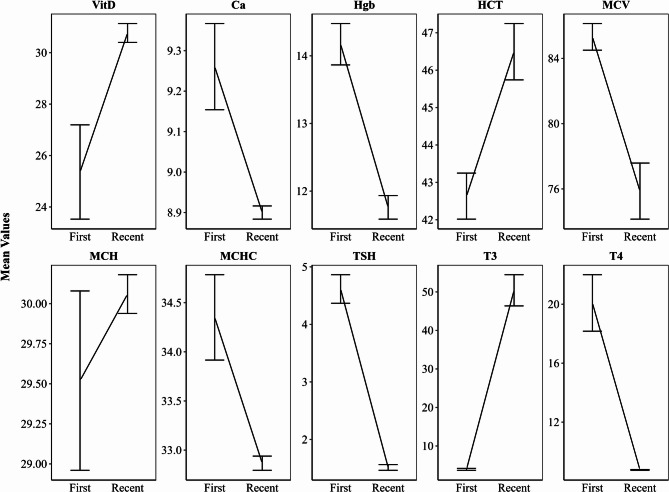
Mean Changes in Laboratory Test Values Over Time in CeD Patients (Displayed as Mean ± Standard Error)

Figure 3 shows changes in laboratory values for CeD patients between their initial and most recent follow-ups. Levels of VitD, HCT, MCH, and T3 increased, while Ca, Hgb, MCV, MCHC, TSH, and T4 decreased. These findings should be interpreted cautiously. Possible explanations include persistent nutrient deficiencies, individual variability in dietary response, or unrelated health factors. These observations highlight the need for further research on long-term metabolic effects of GFD in CeD.

**Fig. 4:**
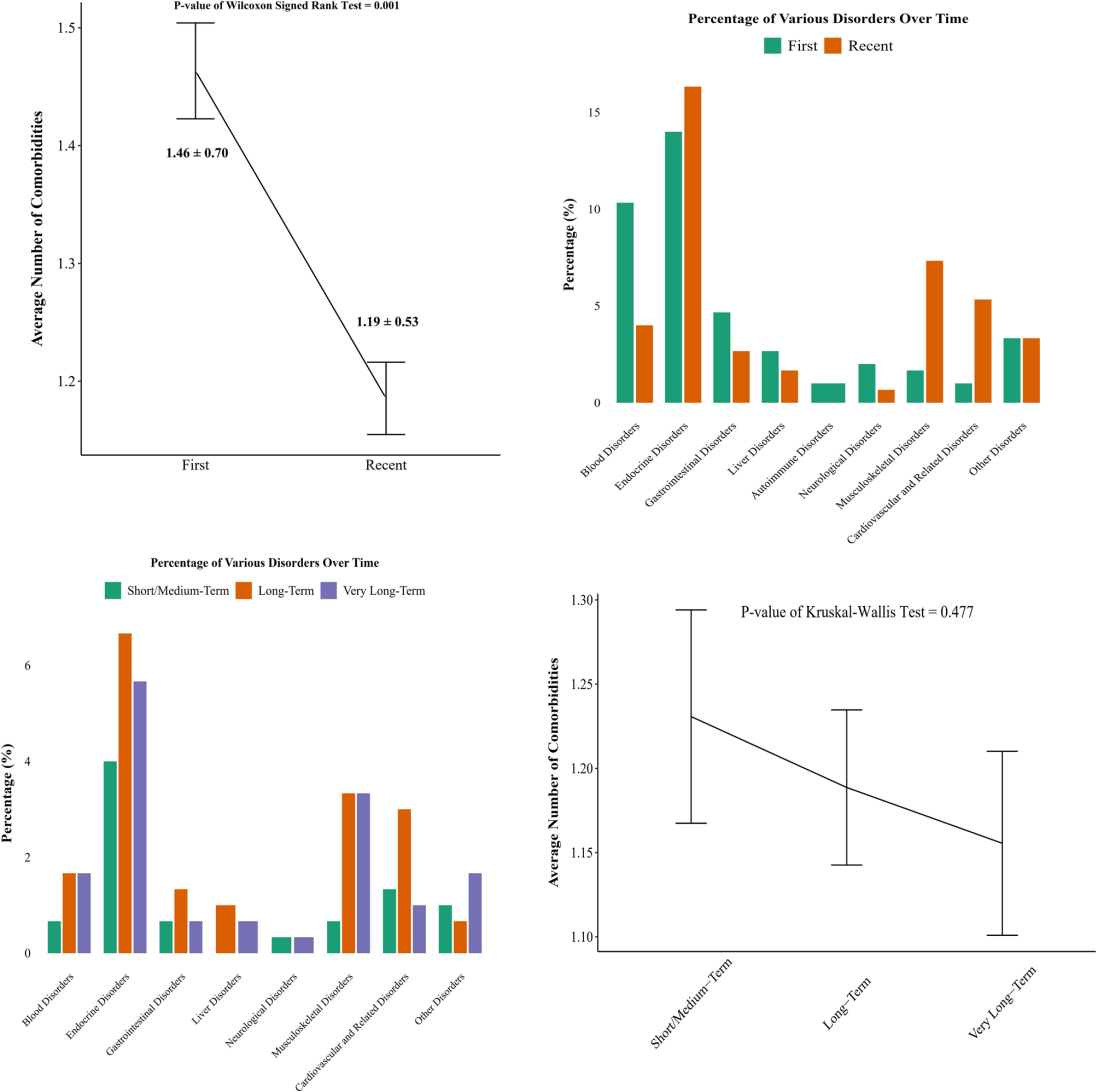
Prevalence of Diagnostic Disorder Categories and Comorbidities at Initial vs. Recent Assessments in CeD Patients, Based on a Distinct Clinical Classification Framework (Line Plot Error Bars Represent Mean ± Standard Error)

Figure 4 expands on this by showing disorder prevalence and comorbidities across assessments and CeD durations. The average number of comorbidities decreased significantly from initial (1.46 ± 0.70) to recent assessments (1.19 ± 0.53, *p* = 0.001). However, no significant differences were observed across CeD duration groups (*p* = 0.477). Disorders like gastrointestinal disorders (other than CeD), liver and cardiovascular related disorders showed reduced prevalence in the very long-term group, reflecting better long-term management.

## Discussion

CeD patients require regular follow-up to assess disease progression, ensure adherence to a strict GFD, and monitor their quality of life as well as their capacity to manage the condition effectively [20, 25–27]. In a study conducted in 2013, Rubio-Tapia and colleagues found that the follow-up of individuals with CeD appears to be less than optimal in practice [28]. In a study conducted in 2018, Çaltepe et al. demonstrated that while diagnostic rates for CeD have improved, a significant number of asymptomatic cases remain undetected. This finding highlights two parallel needs: improved clinical awareness among healthcare professionals to enhance case identification, particularly in high-risk populations, and broader public education to facilitate social integration for diagnosed patients through better societal understanding of GFD requirements [29, 30].

This study sheds light on the diverse clinical profiles of CeD patients, particularly focusing on adherence to a GFD and its implications on health outcomes. One of the primary findings is the substantial variation in GFD adherence, with only 55.33% demonstrating strict adherence as assessed by the Biagi questionnaire. This suggests that most celiac patients may achieve only a gluten-reduced diet in practice, as maintaining the recommended strict GFD proves challenging long-term. The results highlight the tension between clinical ideals and real-world feasibility, as patients struggle with dietary restrictions essential for their health [31]. Unlike previous studies that reported higher adherence rates, our findings underscore a critical gap, where a notable proportion of participants perceived themselves to be adhering to the diet well but were found to have significant errors. This discrepancy reveals the potential for misassessment in self-reported adherence, highlighting the necessity for structured dietary evaluations in clinical settings. This lower adherence rate may reflect regional challenges including limited access to certified gluten-free products in Iran, higher costs relative to local incomes, and fewer dedicated celiac dietitians. In fact, adhering to a GFD can often be challenging for individuals, leading to difficulty maintaining the diet. There are various obstacles they may encounter. These include the risk of cross-contamination between gluten-free products with gluten and poor quality of certified gluten-free products [10]. Accordingly, most patients who were not strictly following a GFD reported that the diet’s difficulty was the main reason for their lapses.

The improvements in both GI and non-GI symptoms observed across the duration of CeD in this study suggest that long-term adherence to a GFD plays a key role in symptom management. According to this finding, it seems that disease management improves over time, likely due to increased awareness and improved clinical care. This finding is in line with previous studies suggesting that early detection and continued adherence to a GFD are pivotal to reduce the long-term burden of CeD [32]. Interestingly, we observed that some symptoms, such as constipation and bloating, did not improve as significantly as others. In some cases, there were slight declines in their absence rates in the very long-term group. The persistent presence of bloating and constipation could indicate that these symptoms might be harder to resolve due to other factors not directly related to gluten intake, such as gut microbiota alterations, which could contribute to symptom persistence. Further research is needed to explore whether other underlying mechanisms, such as dysbiosis or the impact of the GFD on the microbiome, might be influencing these symptoms, as CeD patients often report these issues even after years of GFD adherence.

It is also noteworthy that non-GI symptoms, particularly depression, showed substantial improvement over time. This may reflect psychological normalization following diagnosis, as patients often experience initial distress (anger, fear, or grief) that gradually resolves with GFD adaptation and improved health-related quality of life [15]. Similarly, improving neurologic symptoms and anemia also underscores the beneficial effects of GFD adherence, as these manifestations are often related to nutrient deficiencies or inflammation that can be ameliorated with proper dietary management. Hansen et al. [33] analyzed data from 6,329 Danish patients diagnosed with CeD (2000–2018) and 63,287 matched controls. They found that the incidence of neuropsychiatric disorders was significantly higher in CeD patients, with cumulative incidences increasing over time (3.9–35.9% at different follow-up intervals) compared to controls (1.8–27.0%). The relative risk for developing such disorders in CeD patients was approximately 1.58 times higher. Specific disorders linked to CeD included anxiety, depression, eating disorders, and migraines. Haj Ali et al. [34] investigated the prevalence of anxiety and depressive symptoms among 133 Jordanian CeD patients, utilizing a questionnaire to gather data on demographics and mental health. It found that 85% of participants exhibited anxiety symptoms, while 82.7% had depressive symptoms. Many patients were non-compliant with a GFD, and a significant portion were symptomatic.

The observed changes in laboratory values and comorbidities over time in CeD patients highlight the long-term benefits of strict GFD, including improved disease management and intestinal mucosa healing. The increases in VitD, HCT, MCH, and T3 levels suggest that sustained adherence to the GFD is effective in addressing key deficiencies often seen in CeD patients. These nutrients are vital for overall health and immune function, and their improvement could indicate the positive impact of dietary changes on nutrient absorption and metabolism, especially in the small intestine, which is the primary site of damage in CeD [35].

On the other hand, the decline in Ca, Hgb, MCV, MCHC, TSH, and T4 levels might reflect adjustments in the body as it adapts to dietary changes. This could also imply that certain markers may be sensitive to factors beyond dietary adherence, such as hormonal changes, gut microbiota alterations, or other underlying conditions that influence nutrient absorption and metabolism [36]. The laboratory parameters assessed in our study were selected based on clinical relevance to celiac disease monitoring, data availability in routine follow-up records and known deficiency risks in CeD.

It was observed that the average number of comorbidities significantly decreased over time, from 1.46 ± 0.70 at initial assessments to 1.19 ± 0.53 at more recent assessments. This reduction in the comorbidity burden could reflect improved disease control and a reduction in the systemic inflammation often associated with CeD. Interestingly, no significant differences were found across different CeD duration groups, suggesting that while CeD management generally improves over time, the cumulative duration of the disease may not have a strong effect on the number of comorbidities as initially expected. Furthermore, the lower prevalence of GI disorders (other than CeD), liver, and cardiovascular-related diseases in the very long-term group aligns with findings that prolonged adherence to the GFD can have beneficial effects beyond symptom relief. Despite these positive trends, the absence of significant differences across CeD duration groups suggests that other factors, such as patient education, access to healthcare, and adherence to follow-up care, may also play crucial roles in managing these comorbidities.

This study has some limitations that should be acknowledged. First, our study’s reliance on retrospective data precludes definitive conclusions about causality. The observed associations between GFD adherence and health improvements may also reflect other factors, such as natural disease progression or regression. Additionally, the absence of a control group (either untreated CeD patients or healthy individuals) limits our ability to determine whether observed improvements reflect true dietary effects rather than natural fluctuations, aging, or other temporal factors.

Second, our reliance on patient-reported outcomes introduces potential recall bias and social desirability bias in dietary reporting. While we used the validated Biagi questionnaire, the observed discrepancy between self-perceived and scored adherence confirms this limitation.

Third, while the Biagi questionnaire provided a validated measure of strict GFD compliance, future studies could benefit from incorporating multi-dimensional adherence tools (e.g., CDAT, SDE) to capture dietary behaviors, cross-contamination risks, and psychosocial factors more comprehensively. Our adherence assessment did not include detailed dietary recalls, which may provide finer detection of cross-contamination.

Forth, our 23.7% response rate (300 of 1268) is modest, but this is common for phone surveys in medical research, especially for chronic conditions like CeD. Several factors contributed to this. For instance, many patients had changed contact details years after diagnosis and some were unavailable during study hours. However, it may introduce selection bias. Furthermore, excluding initial non-adherers subjects means our findings primarily reflect outcomes in diet-attempting patients, potentially underestimating challenges in the most resistant cases.

Fifth, while our study population was predominantly female (73.67%), reflecting the well-documented gender disparity in CeD diagnosis and clinical follow-up, we did not identify overt differences in GFD adherence or symptom trajectories between sexes during preliminary data review. However, our findings may disproportionately represent female experiences. Moreover, while we controlled for key clinical variables, unmeasured confounders including socioeconomic status, regional access to gluten-free products, and health literacy may partially explain observed adherence patterns and symptom improvements.

Sixth, our reliance on symptom-based outcomes and basic laboratory measures, rather than standardized endoscopic, serological, or psychometric evaluations, may limit the robustness of our findings. While we observed improvements in self-reported depression and comorbidity burden, these were not assessed using validated scales or uniformly verified through medical records. Similarly, the absence of routine follow-up biopsies or serial serological testing prevents definitive conclusions about mucosal healing and immunological response. The nutritional adequacy of participants’ diets was not evaluated, limiting our ability to correlate lab changes with specific dietary patterns or supplement use. Our inability to account for medication or supplement use represents a key limitation, particularly when interpreting laboratory trends (e.g., Ca, Hb, MCV changes).

## Conclusions

This study of 300 celiac patients highlights the importance of ongoing, personalized monitoring to assess both dietary adherence and evolving health needs. Our findings demonstrate that multidisciplinary care involving dietitians, gastroenterologists, and mental health professionals are essential to facilitate informed dietary choices, manage comorbidities, and improve overall quality of life. Given that physicians were the primary source of dietary information for patients in our study, improving clinician education on CeD management could help optimize adherence support. Future research should directly evaluate healthcare provider knowledge gaps and their impact on patient outcomes.

## Supplementary Information

Supplementary Material 1.

## Data Availability

The datasets supporting the conclusions of this article are included within the article and its additional file.

## Abbreviations

AGA: American Gastroenterological Association
BMI: Body mass index
CeD: Celiac disease
GFD: Gluten-free diet
GI: Gastrointestinal
IQR: Interquartile range
SD: Standard deviation
