# Supplementary material for: Gluten-free diet adherence patterns and health outcomes in celiac disease: a retrospective observational study
Source: BMC Gastroenterol. 2025 Aug 18;25:591. doi: 10.1186/s12876-025-04193-3 (PMC12359905; doi:10.1186/s12876-025-04193-3)
Supplement: Supplementary file 1 — Supplementary Material 1. [file 12876_2025_4193_MOESM1_ESM.pdf]

celiac disease patients’ follow-up

<table>
<tr><td colspan="3" rowspan="2">Name :</td><td>Age:</td><td rowspan="2">Sex:
Male □ Female □</td><td colspan="2" rowspan="2">Marriage:
Single□ Married□
widow□ Divorced□</td></tr>
<tr><td>Number of children:</td></tr>
<tr><td colspan="3" rowspan="2">Race:</td><td>Celiac disease duration:</td><td rowspan="2">Education:</td><td colspan="2" rowspan="2">Job:</td></tr>
<tr><td>GFD duration:</td></tr>
<tr><td rowspan="6">Smoking:</td><td rowspan="4">Yes</td><td rowspan="2">Previously</td><td rowspan="4">Date of first diagnosis:</td><td rowspan="6">Family history of celiac disease:
Yes □ No □ I Don’t know□

Family relationship:
First degree□
Second degree□</td><td rowspan="2">Height</td><td>At the time of diagnosis</td></tr>
<tr><td>Currently</td></tr>
<tr><td>Currently</td><td rowspan="2">Weight</td><td>At the time of diagnosis</td></tr>
<tr><td>Sometimes</td><td>Currently</td></tr>
<tr><td colspan="2" rowspan="2">No</td><td>History of other diseases at the time of diagnosis:</td><td rowspan="2">BMI</td><td>At the time of diagnosis</td></tr>
<tr><td>Have you been diagnosed with any other disease during the period of celiac disease? Which one:</td><td>Currently</td></tr>
</table>

- What was your tTG IgA level at the time of diagnosis?
- What was the severity of your intestinal injury at the time of diagnosis (according to the Marsh classification)?
- Which of the following tests have you done recently?

IgA EMA: Result……... IgA tTG: Result……… IgA AGA: Result…… IgG AGA: Result……. Total IgA: Result……

- Have you done endoscopy and pathology evaluation recently? Result........

Gastrointestinal symptoms

- Which of the following symptoms did you have **at the time of diagnosis**?
  Diarrhea □ nausea and vomiting □ Weight loss □ Abdominal pain □ constipation □
  Bloating □ stomach cramps □ Gas feeling □ Passing gas □ Fatigue□
- Which of the following symptoms do you **currently** have?

  Diarrhea □ nausea and vomiting □ Weight loss □ Abdominal pain □ constipation □
  Bloating □ stomach cramps □ Gas feeling □ Passing gas □ Fatigue□

Extra-intestinal symptoms

- Which of the following symptoms did you have **at the time of diagnosis**?

Anemia □ (which type?.........)

bone problems □ (bone pain □ Osteoporosis□ Bone fracture□)

Muscle weakness□ muscle pain□ Neurological symptoms□ History of depression□

History of stress□ Menstrual disorders□ Infertility □ Abortion□ Aphthous□

Dermatologic Manifestations□ Dental enamel problems □

- Which of the following symptoms do you **currently** have?

Anemia □ (which type?.........)

bone problems □ (bone pain □ Osteoporosis□ Bone fracture□)

Muscle weakness□ muscle pain□ Neurological symptoms□ History of depression□

History of stress□ Menstrual disorders□ Infertility □ Abortion□ Aphthous□

Dermatologic Manifestations□ Dental enamel problems □

- Complete checkup results **at the time of diagnosis**

Alb: Creat: Fe: Folate: Ferritin: VitD Ca: Mg: ALP: ALT:
AST: Hgb: HCT: MCV: MCH: MCHC: BiL-T: BiL-D: B12:
TG: Chol: TSH: T3: T4: FBS:

- **Recent** full checkup results

Alb: Creat: Fe: Folate: Ferritin: VitD Ca: Mg: ALP: ALT:
AST: Hgb: HCT: MCV: MCH: MCHC: BiL-T: BiL-D: B12:
TG: Chol: TSH: T3: T4: FBS:

- In your opinion, how do you follow the gluten-free diet (GFD)?

I do not follow the diet □ I follow the diet intermittently □ I follow the diet strictly□

- How has your GFD adherence changed in recent years compared to the early years of the disease diagnosis?

It has become poorer□ It has become better□ It does not change□

- What is the reason if compliance with the diet becomes poorer?

Financial problems□ The difficulty of the diet□ Lack of easy access to gluten-free products□

- What are the main gluten-containing foods that you have to use? Name it

- How to follow the diet based on Biagi's questionnaire

<table>
<tr><td colspan="4">Do you eat gluten voluntarily?</td></tr>
<tr><td colspan="3">Yes</td><td rowspan="4">No</td></tr>
<tr><td rowspan="3">How much?</td><td colspan="2">A normal portion</td></tr>
<tr><td rowspan="2">Just a taste</td><td>Often</td></tr>
<tr><td>Rarely</td></tr>
<tr><td colspan="4">When you eat out, do you tell the person who is cooking about your disease?</td></tr>
<tr><td colspan="3">Yes</td><td>No</td></tr>
<tr><td colspan="4">Do you check the labels of packaged food?</td></tr>
<tr><td colspan="3">Yes</td><td>No</td></tr>
<tr><td colspan="4">Do you only eat packaged food guaranteed by the coeliac Association</td></tr>
<tr><td colspan="3">Yes</td><td>No</td></tr>
</table>

- How did you get your information about how to follow the diet correctly?
  Social media□ Consultation with a physician and nutritionist□ From books and catalogs□ Asking questions of affected people□
- Have you gotten infected with COVID-19? Yes□ No□
- What was the severity of your COVID-19 infection? weak □ moderate□ severe□
- Have you injected the COVID-19 vaccine? Which type?
- Have you had an unsuccessful marriage during your disease period? What was the reason?
